# Supplementary material for: Direct Visualization of Amlodipine Intervention into Living Cells by Means of Fluorescence Microscopy
Source: Molecules. 2021 May 18;26(10):2997. doi: 10.3390/molecules26102997 (PMC8158129; doi:10.3390/molecules26102997)
Supplement: Supplementary file 1 [file molecules-26-02997-s001.zip › molecules-1217895-SUPPL/molecules-1217895-suppl.pdf]

Supplementary Materials

Direct Visualization of Amlodipine Intervention into Living Cells by Means of Fluorescence Microscopy

Christine Quentin [1], Rūta Gerasimaitė [2], Alexandra Freidzon [3,4], Levon S. Atabekyan [3], Gražvydas Lukinavičius [2], Vladimir N. Belov [1] and Gyuzel Y. Mitronova [1,*]

[1] Department of NanoBiophotonics, Max Planck Institute for Biophysical Chemistry, Am Fassberg 11,
37077 Göttingen, Germany; christine.quentin@mpibpc.mpg.de (C.Q.); vladimir.belov@mpibpc.mpg.de (V.N.B.)

[2] Chromatin Imaging and Labeling Group, Department of NanoBiophotonics, Max Planck Institute for Biophysical Chemistry, Am Fassberg 11, 37077 Göttingen, Germany; ruta.gerasimaite@mpibpc.mpg.de (R.G.); grazvydas.lukinavicius@mpibpc.mpg.de (G.L.)

[3] Federal Research Center Crystallography and Photonics, Photochemistry Center, Russian Academy of Sciences, Novatorov 7a, 119421 Moscow, Russia; freidzon.sanya@gmail.com (A.F.); levat51@mail.ru (L.S.A.)

[4] National Research Nuclear University MEPhI (Moscow Engineering Physics Institute), Kashirskoye Shosse 31, 115409 Moscow, Russia

* Correspondence: gyuzel.mitronova@mpibpc.mpg.de

Table of Contents

Supplementary Results 4

Supplementary Figures 4

Figure S1. Fluorescence enhancement of AML in protein solutions. 4

Figure S2. Absorption and fluorescence spectra of AML in PBS and PBS containing 0.1% sodium dodecyl sulfate (SDS) or 10% BSA 5

Figure S3. Solvatochromic behavior of AML. 6

Figure S4. Quantum chemical calculations. 7

Figure S5. Viscosity dependence of AML fluorescence. 8

Figure S6. Laser-flash photolysis (LFP) and fluorescence measurements of 10 μM AML in buffer solution and in 10% BSA solution. .... **Error! Bookmark not defined.**

Figure S7. AML photobleaching in live A7r5 cells. 10

Figure S8. Glyoxal fixed HEK-293 $Ca_V1.2$ cells and HEK-293 cells immunostained for $Ca_V1.2$. 11

Figure S9. Comparison of AML staining on HEK-293 (no CaV1.2 expression) and HEK-293 CaV1.2 overexpressing cells, uninduced and after one day doxycycline induction (HEK CaV1.2 + dox). 12

Figure S10. Confocal images showing live A7r5 cells co-stained with AML and cellular marker 13

Figure S11. pH dependence of AML emission. 14

Figure S12. FLIPR 6 Ca^{2+} assay (a) and dose-response curves of AML and (*S*)-AML (b) obtained on HEK-293 $Ca_V1.2$ cells and HL-1 cells. 15

Supplementary Movies, description 16

Supplementary Table 17

Table S1. Atomic coordinates of the model AML in the equilibrium ground S_0, first excited S_1 and transition states as well as in the conical intersection between the S_1 and S_0 states. 17

Table S2. Calculated absorption and emission spectra of AML and AML-5-COOH 24

Synthesis of AML-5-COOH 25

General materials and methods. 25

Compound 1 25

Compound 2 26
Compound 3 26
Compound 4 26
Compound 5 27
Compound 6 27
AML-5-COOH 28
Abbrevations 30
References 31

Supplementary Results

Supplementary Figures

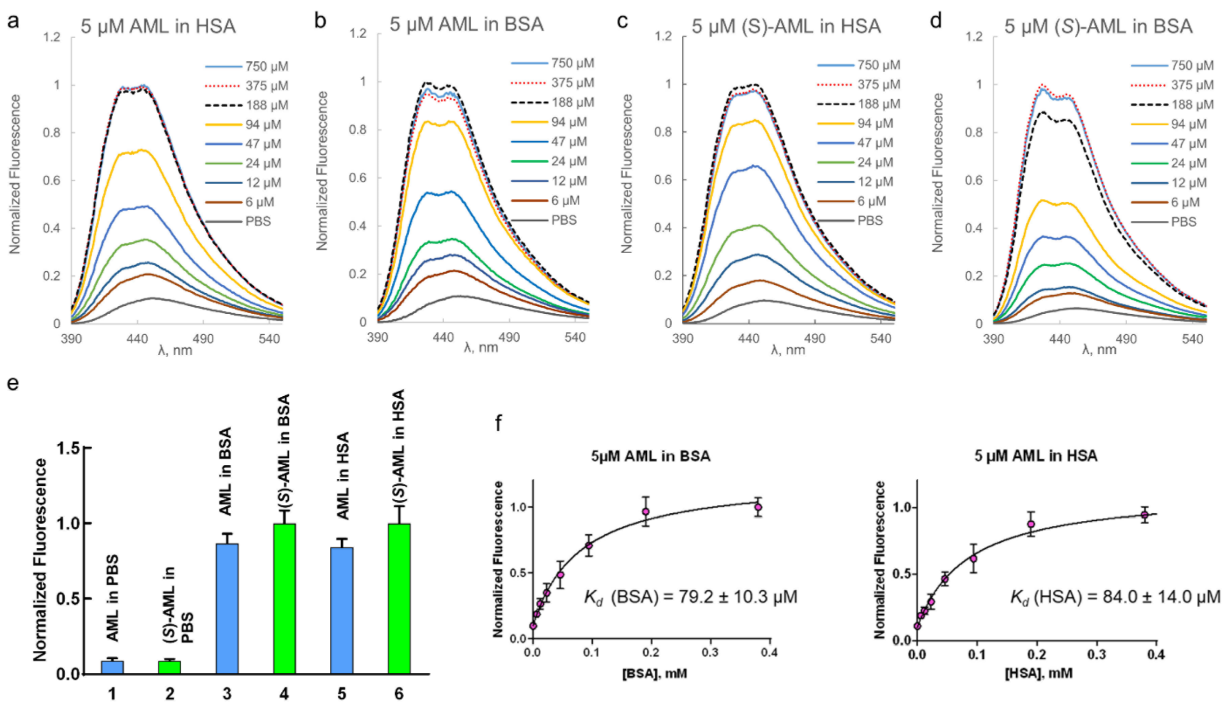

Figure S1. Fluorescence enhancement of AML in protein solutions. Upon AML binding to the BSA/HSA hydrophobic pocket, the fluorescence intensity increases. Fluorescence spectra of 5 μM (a,b) AML or (*S*)-AML (c,d) in HSA/BSA**.** Incubation 1 h. The spectra have been recorded in triplicate in black polystyrene 96 well plates (Mattek; transparent, flat bottom 96 well plate) on a Spark 20M (Tecan) microplate reader at 25 °C. Excitation wavelength 360 nm, excitation bandwidth 7.5 nm, emission bandwidth 10 nm, gain 100, number of flashes 20. The fluorescence intensity scans were measured from 390 to 550 nm. The background was subtracted, and fluorescence normalized to the mean AML/(*S*)-AML signal saturation protein concentration. (e) Fluorescence emission enhancement upon of formation of AML-BSA or AML-HSA complexes after 1 h incubation of 5 μM AML/(*S*)-AML in 375 μM BSA/HSA at rt; the emission intensity at the maximum of 451 nm were measured. Mean values ± S.D. are shown (n=3). (e) Binding of AML to BSA and HSA measured by titrating 5 μM AML with 6 μM to 0.38 mM BSA/HSA. Values are mean ± SD, n=3 independent measurements. Dissociation constants (K_d, mean ± S.E.M.) were computed using the software package GraphPad Prism version 8.3.1.

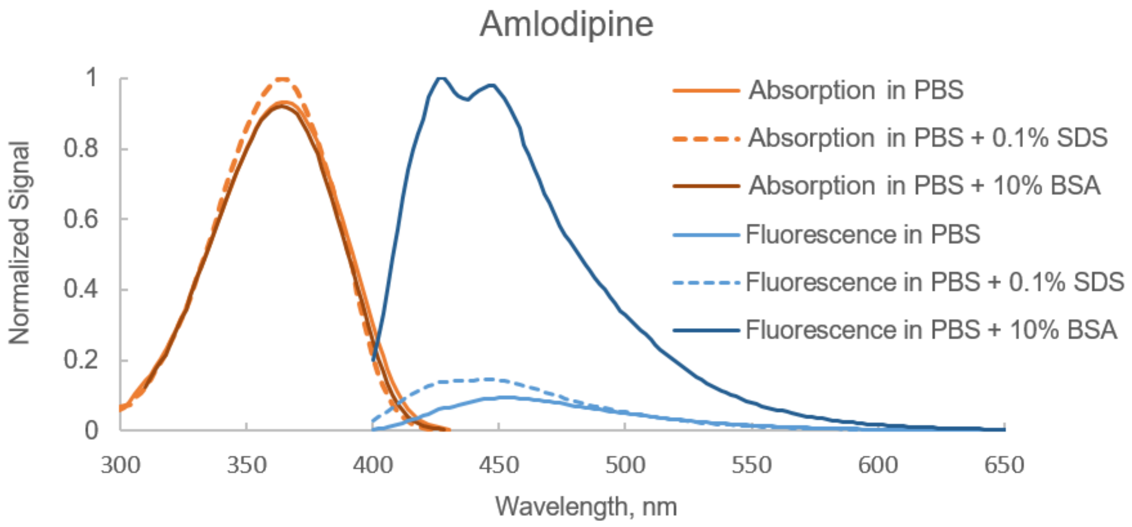

Figure S2. Absorption and fluorescence spectra of AML in PBS (pH 7.4 + 0.1 v/v% DMSO) and PBS containing 0.1% sodium dodecyl sulfate (SDS) or 10% BSA. The addition of anionic surfactant (SDS) had little effect on AML fluorescence intensity indicating that self-aggregation of AML is not responsible for the emission enhancement. The absorption spectra were recorded on a Varian Cary 4000 UV-Vis spectrophotometer in quartz cuvettes with a 1 cm path length. The fluorescence spectra of AML have been recorded on a Varian Cary Eclipse fluorescence spectrometer, excitation 365 nm.

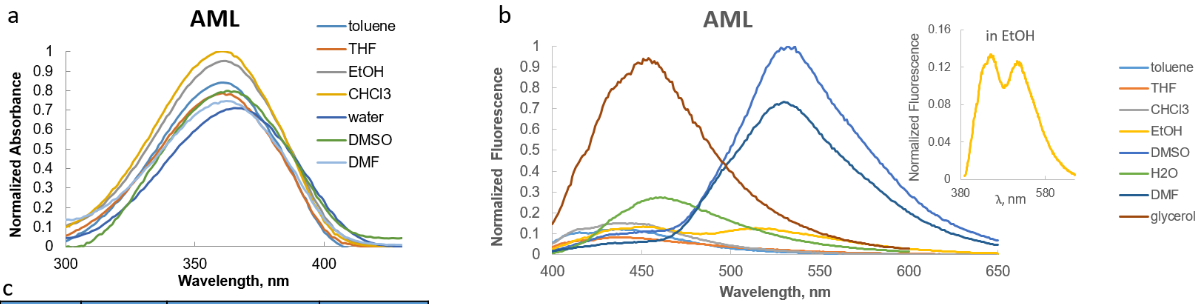

c

	λ_{em}, nm	Viscosity(η), mPa × s	Dielectric constant
**toluene**	436; 413	0.59*	2.4*
**CHCl3**	439; 416	0.58*	4.81*
**THF**	440, 416	0.55*	7.6*
***n*-octanol**	513; 453	10.6*	10.3
***n*BuOH**	513; 453	2.95*	17.8*
***i*PrOH**	509; 454	2.86*	18.3*
**aceton**	441	0.32*	20.7*
**EtOH**	518; 451	1.25*	24.55*
**MeOH**	455	0.55*	32.6*
**DMAA**	532	2.14*	37.8
**DMF**	532	0.92*	36.7
**glycerol**	455	945**	42.5**
**DMSO**	534	1.996**	46.7**
**H_2O**	463	1.00*	78.5*
**Formamide**	461	3.3**	109.5**

*https://www.sigmaaldrich.com/content/dam/sigma-aldrich/docs/Aldrich/General_Information/labbasics_pg144.pdf
**https://www.trimen.pl/witek/ciecze/old_liquids.html

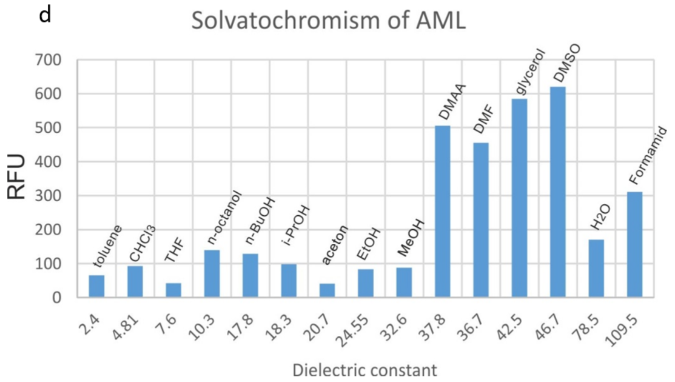

Figure S3. Solvatochromic behavior of AML. The fluorescence enhancement is stronger in polar, aprotic solvents and glycerol. Absorption (a) and emission (b) spectra were acquired in solvents of different polarity, H-bonding capabilities and viscosities. (c) Positions of emission maxima of AML in different solvents, viscosities and dielectric constants of solvents. (d) Fluorescence maxima *vs* polarity of solvents. The fluorescence enhancement is detected in polar, aprotic solvents and in viscous glycerol.

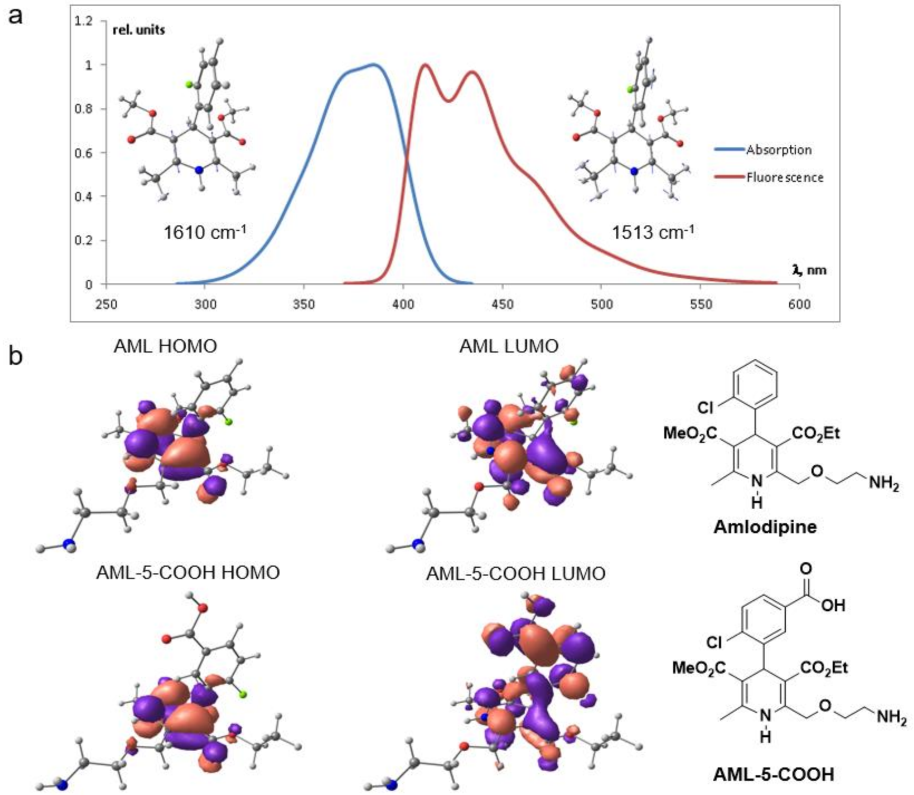

Figure S4. Quantum chemical calculations. (a) Calculated absorption and emission spectra of model DHP. (b) Molecular orbitals of AML and AML-5-COOH. The both electron density of the highest occupied (HOMO) and lowest unoccupied (LUMO) molecular orbitals of AML are localized mainly on DHP, while in AML-5-COOH, containing the electron-withdrawing substituent COOH, LUMO is localized on both DHP and aromatic ring. The vibronic absorption and emission spectra of dimethyl 4-(2-chlorophenyl)-2,6-dimethyl-1,4-dihydropyridine-3,5-dicarboxylate were calculated according to the Lax model[1, 2] utilizing the time-domain formalism and the multi-mode harmonic oscillator model.[3-6] Molecular orbitals of AML and AML-5-COOH calculated by PBE0/6-31+G(d,p). Color denotes positive and negative orbital phase.

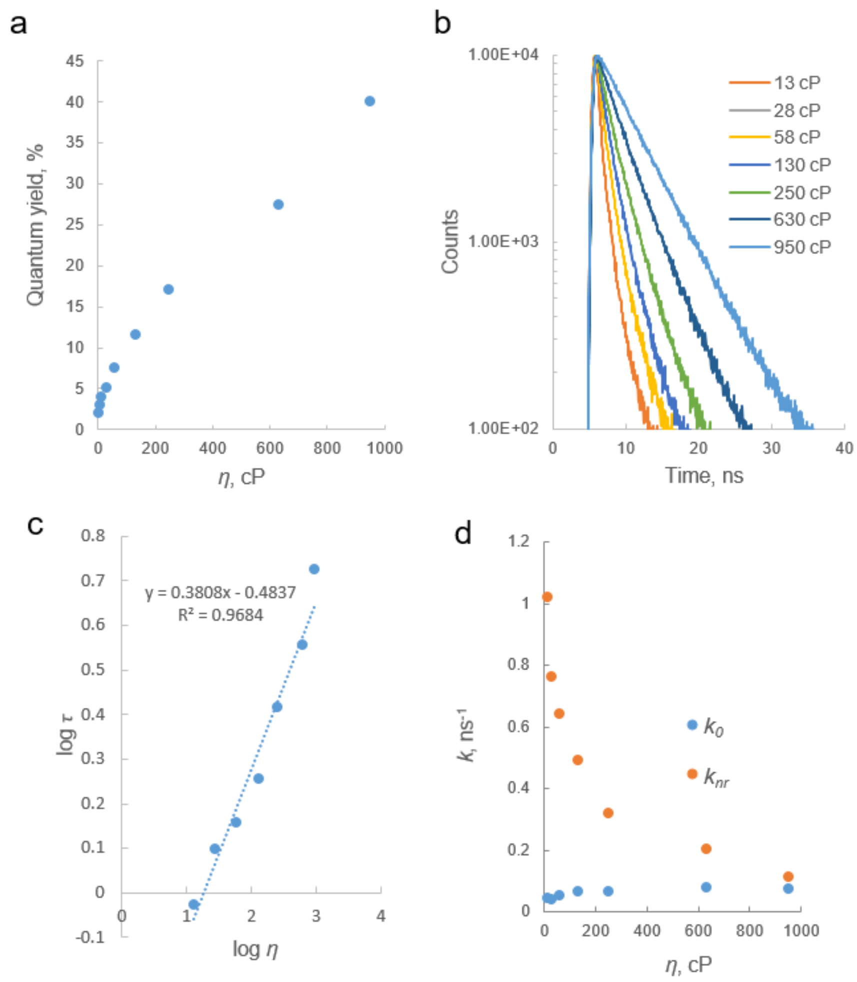

Figure S5. Viscosity dependence of AML fluorescence. (a) Fluorescence quantum yield of AML as a function of solvent viscosity (η). (b) Decay traces of AML. (c) Overall recorded fluorescence lifetime (τ) versus solvent viscosity (η) plotted on a logarithmic scale. (d) Non-radiative (k_{nr}) and radiative (k_0) decay constants of AML calculated according to: $\Phi = \frac{k_0}{k_0+k_{nr}}$ and $\tau = \frac{1}{k_0+k_{nr}}$, where Φ – fluorescence quantum yield and τ – fluorescence lifetime. k_0 remains almost constant and decreasing k_{nr} with increasing viscosity of the medium. Thus, partial constraining of AML molecular twisting and rotations by a viscous medium prevents its transition to a non-emissive "dark" state.

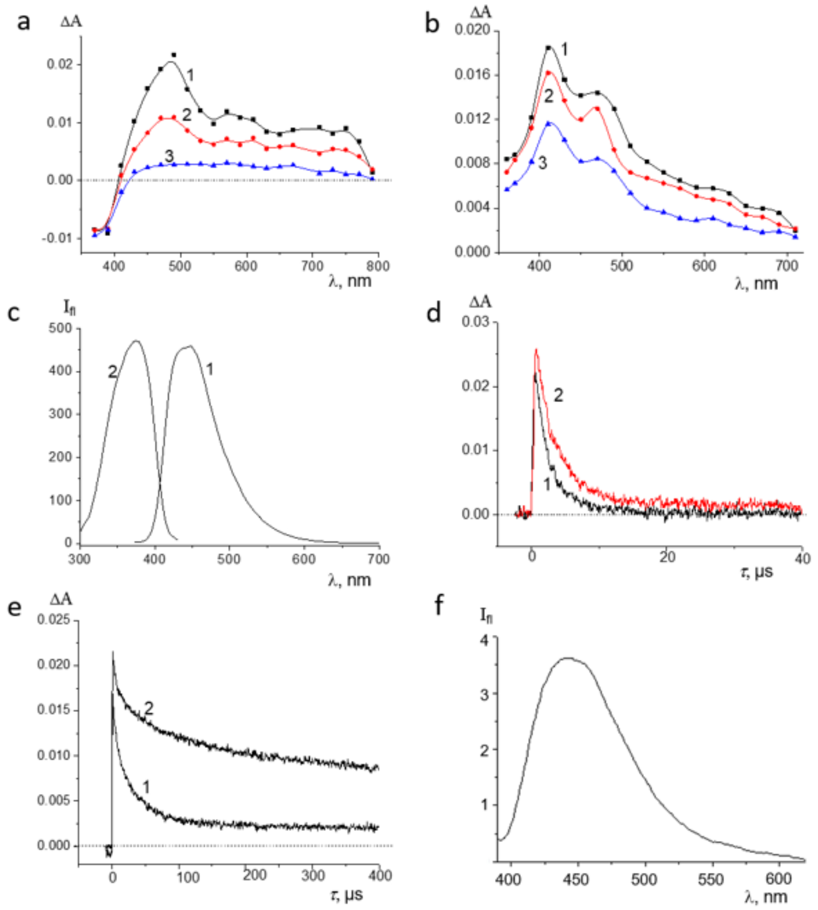

	PBS	PBS +10% BSA
k_1 (s^{-1})	$7\times10^{5*}$ $3\times10^{5**}$	$1\times10^{5*}$ $8\times10^{4**}$
k_2 (s^{-1})	$2\times10^{5*}$ $5\times10^{4**}$	$2\times10^{5*}$ $7\times10^{3**}$

* air-saturated; ** deoxygenated.

Figure S6. Laser-flash photolysis (LFP) and fluorescence measurements of 10 μM AML in buffer solution and in 10% BSA solution. (a) Photoinduced time resolved transient absorption spectra of the oxygen-free AMD solution in PBS measured 1 μs (1), 3 μs (2), and 10 μs (3) after the end of the laser pulse. (b) Photoinduced time resolved transient absorption spectra of the oxygen-free AMD solution in PBS containing 10 % BSA measured 1 μs (1), 3 μs (2), and 10 μs (3) after the end of the laser pulse. (c) Fluorescence spectrum (1) and fluorescence excitation spectrum (2) of AML in 10% BSA in PBS. Spectra are measured after 1 h incubation at rt. (d) Kinetic curves of the photoinduced absorption of AMD in PBS, measured at 510 nm for air-saturated (1) and deoxygenated (2) solutions. (e) Kinetic curves of the photoinduced absorption of AMD in PBS containing 10% BSA, measured at 430 nm for air-saturated (1) and deoxygenated (2) solutions. The relaxation kinetics of the induced absorption is bi-exponential with the rate constants (*k*) represented in the Table. (f) Delayed fluorescence spectrum of AML in PBS containing 10% BSA measured in the phosphorescence measurement mode (delay 200 μs). λ_{exc} = 365 nm. The spectrum is similar to its prompt fluorescence spectrum, indicating the presence of a non-emissive triplet state.

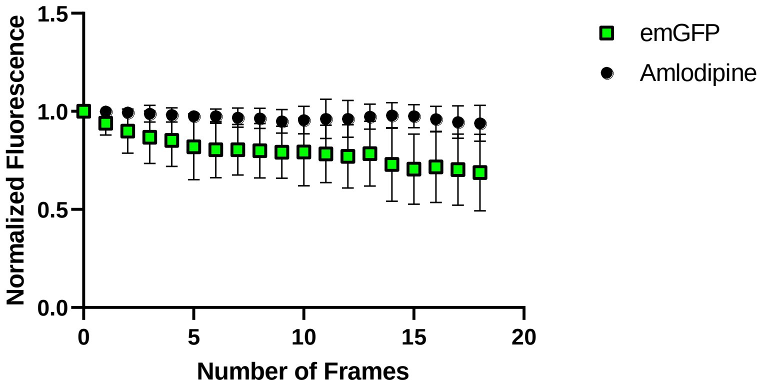

Figure S7. AML photobleaching in live A7r5 cells. Bleaching experiments show less photobleaching of AML compared to the emGFP. The living A7r5 cells expressing LAMP1-emGFP were treated with 300 nM AML (15 min incubation at 37°C) in DMEM, washed twice with DMEM. Time-lapse images were then acquired on Leica SP8 confocal microscope. 5% laser power for 405 nm and 488 nm lines was used. Error bars represent the standard error across data obtained from 4 cells per trace.

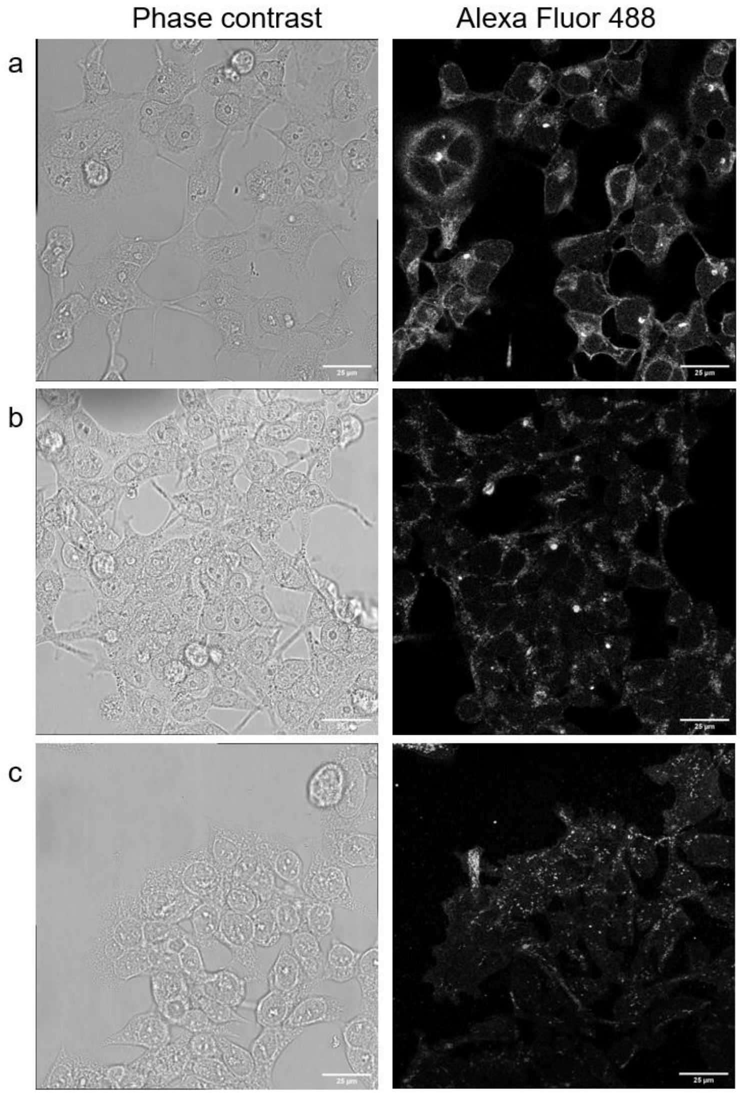

Figure S8. Glyoxal fixed HEK-293 Cav1.2 cells and HEK-293 cells immunostained for Cav1.2. The labelling pattern is markedly different from the AML staining (see for example Figure S9). a) The Cav1.2 expression was induced with 0.1 μg/ml doxycycline (1 day). The cells are remarkable enlarged compared to uninduced cells (b); some of them appear as multinucleated giant cells; b) uninduced HEK-293 Cav1.2 cells; c) HEK-293 cells. Anti-Cav1.2 (CACNA1C) antibody (rabbit polyclonal, Alomon labs) coupled to Alexa Fluor 488 were used. The glyoxal fixation was performed according to Richter et al.[7] The images were acquired on SP8 confocal microscope using settings as described in the Methods section.

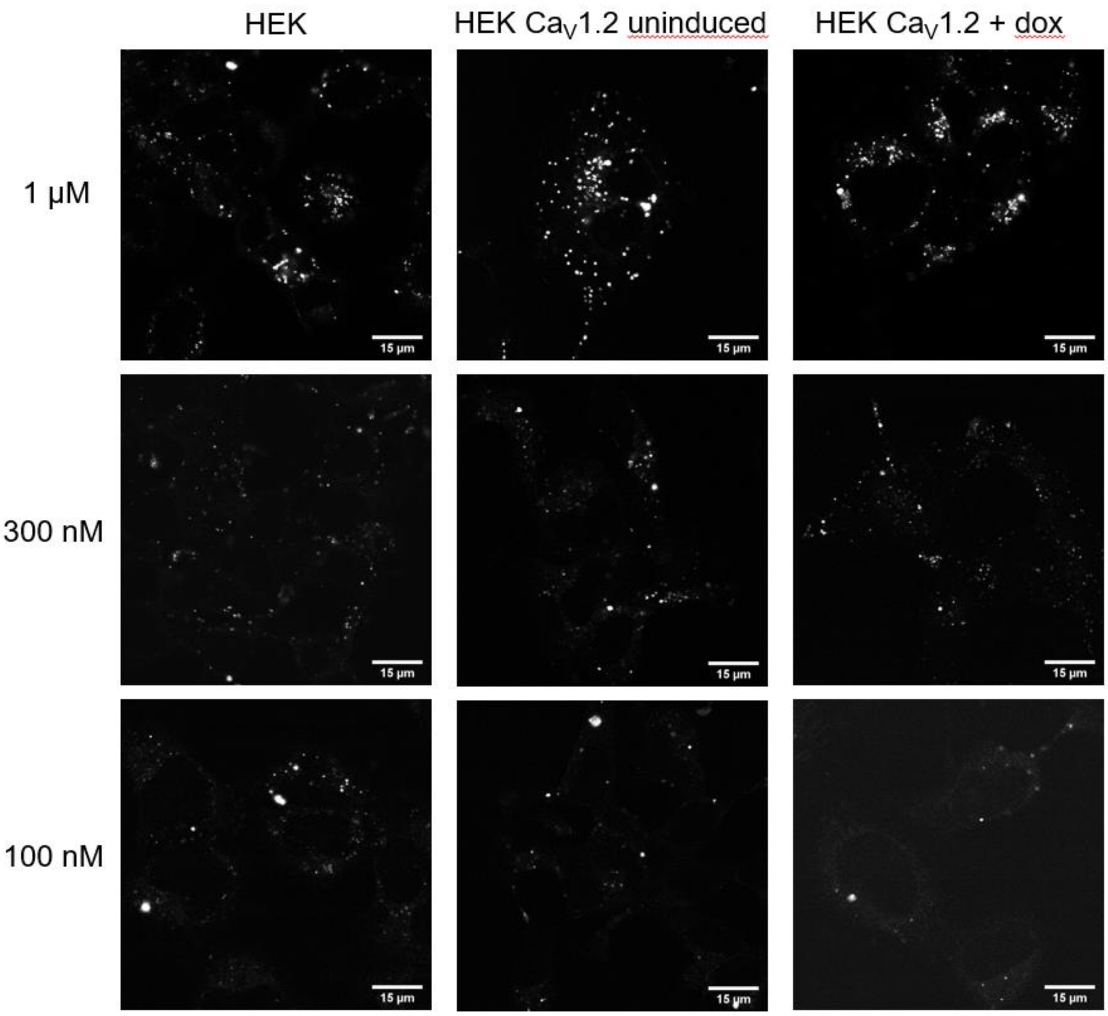

Figure S9. Comparison of AML staining on HEK-293 (no Cav1.2 expression) and HEK-293 CaV1.2 overexpressing cells, uninduced and after one day doxycycline induction (HEK Cav1.2 + dox). The staining efficiency is similar in all cells indicating that internalization of AML does not depend on the interaction with Cav1.2. Cells were incubated for 1h at 37°C in HBSS/HEPES containing indicated concentrations of ligands, washed with HBSS and imaged in HBSS on confocal SP8 microscope using settings as described in the Methods section.

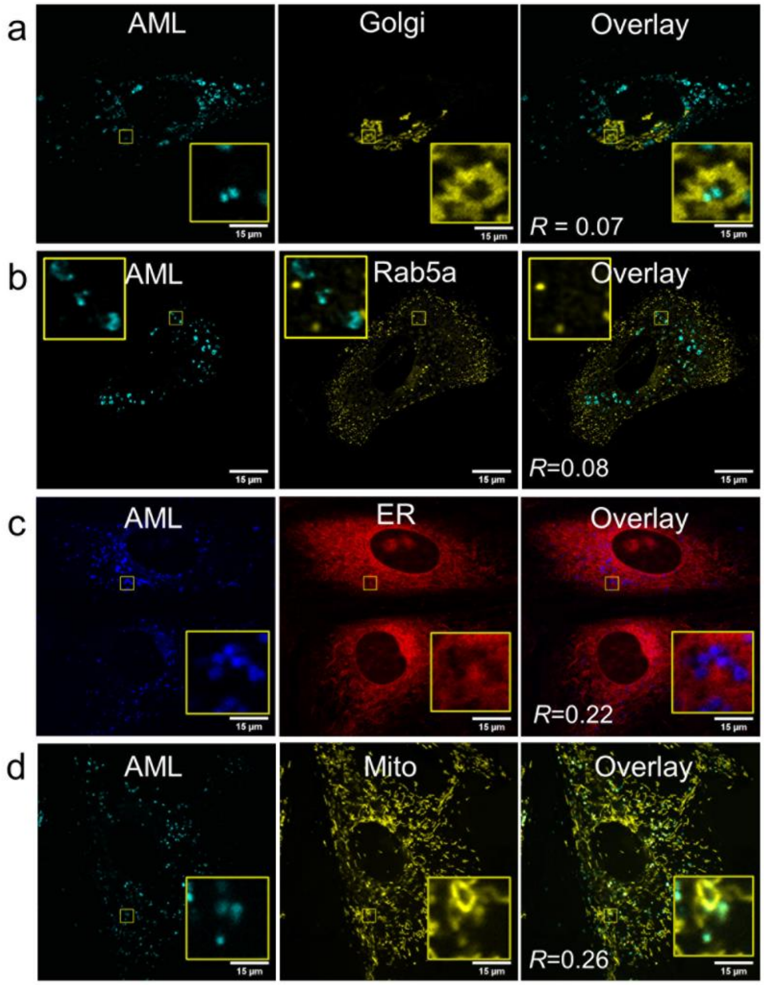

Figure S10. Confocal images showing live A7r5 cells co-stained with AML (cyan or blue) and cellular markers (yellow or red). (a) CellLight® Golgi-emGFP, BacMam 2.0 for the labelling of Golgi, (b) CellLight® Rab5a-emGFP, BacMam 2.0 for the labelling of early endosomes, (c) ER-Tracker® Red for the labelling of ER, (d) MitoTracker® Orange (100 μM) for the labelling of mitochondria. AML staining displays no or weak correlation with these organelles. The cells were incubated in HBSS supplemented with 20 mM HEPES (pH 7.4), then washed twice and imaged on confocal Leica SP8 microscope as described in the Materials and Methods section. Incubation (37 °C) time for AML– 1h, all other trackers – according to the manufacturer's procedures. Whole cells and selected ROIs 5×5 μm are shown. Pearson's co-localization coefficients above threshold (*R*) were determined using Huygens Essential version 20.10.

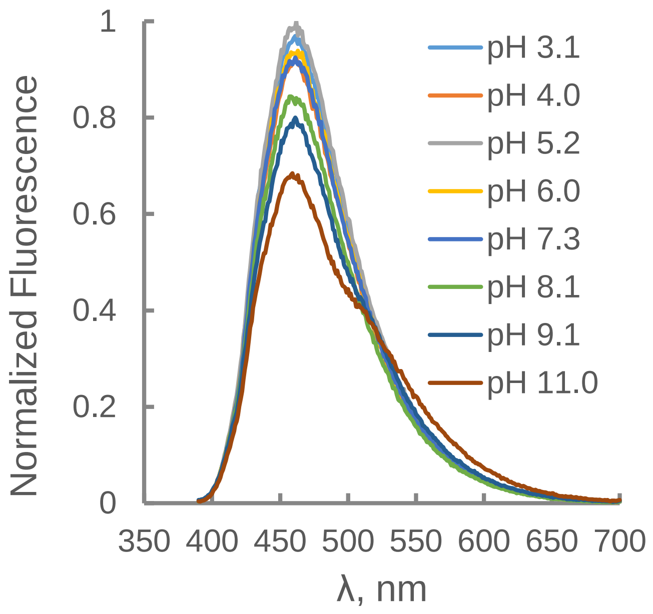

Figure S11. pH dependence of AML emission. The fluorescence of AML at cytoplasmic pH levels (~7.0) is slightly lower than at the pH values corresponding to endosomal or lysosomal lumen (~5.0), maintaining > 90% of its maximal fluorescence intensity. Fluorescence spectra of 5 μM AML in PBS solutions of different pH (3 – 9) were recorded on a Varian Cary Eclipse fluorescence spectrometer.

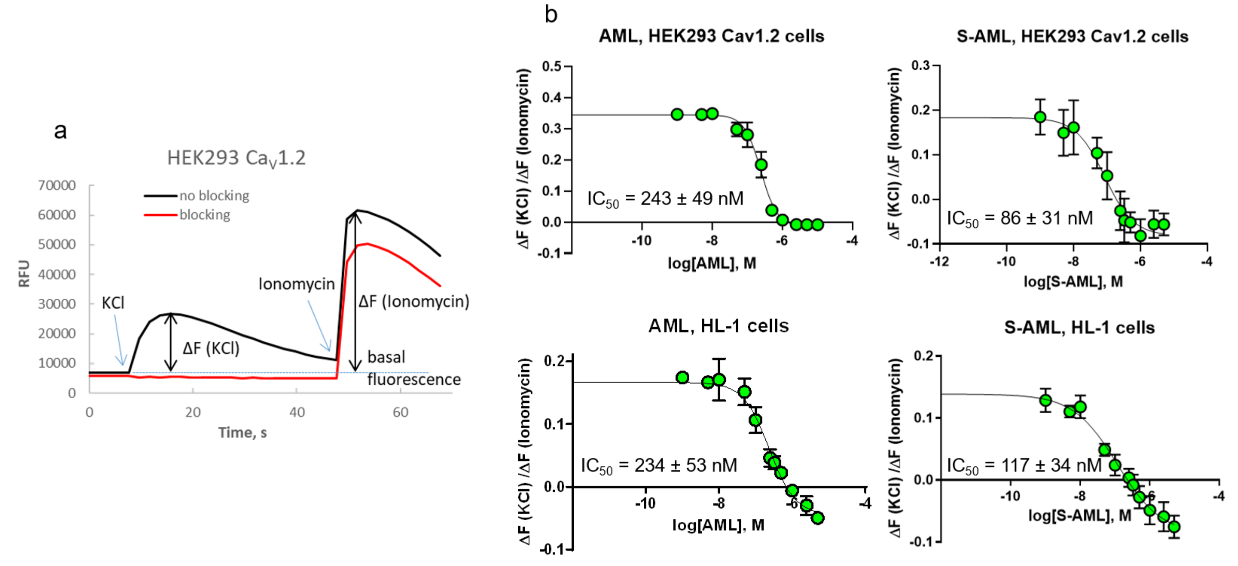

Figure S12. FLIPR 6 Ca^{2+} assay (a) and dose-response curves of AML and (*S*)-AML (b) obtained on HEK-293 Cav1.2 cells and HL-1 cells. In the concentrations used in the study, AML acts as a long-lasting Ca^{2+} channel blocker. The response on KCl addition, ΔF (KCl), was normalized to the maximal response on ionomycin addition, ΔF (Ionomycin). The data represent as mean ± SD, n = 4–8 independent measurements. IC_{50} (mean ± SD) were calculated using the software package GraphPad Prism version 8.3.1.

Supplementary Movies, description

Movie S1. 3D confocal time-lapse images of direct amlodipine loading (300 nM, magenta) on live A7r5 cells stained with lysosomal marker LAMP1-GFP (green), co-localized pixels appear in white. The z-stacks were obtained each minute (duration 18 min) on SP8 confocal microscope using settings as described in the Methods section.

Movie S2. Z-stack of confocal images of live A7r5 co-stained with 300 nM Amlodipine (magenta), lysosomal marker LAMP1-GFP (green).

Movie S3. Confocal time-lapse images of live A7r5 co-stained with 300 nM Amlodipine (blue), lysosomal marker LAMP1-GFP (green) and 5μM membrane marker FM 4-64 (red). The images were obtained in the line sequential mode after 30 min incubation. Frame – 6 s, duration 3 min.

Movie S4. Confocal time-lapse images of live A7r5 co-stained with 300 nM Amlodipine (blue), lysosomal marker LAMP1-GFP (green) and 5μM membrane marker FM 4-64 (red). Co-localized pixels appear in pink. The images were obtained in the line sequential mode after 180 min incubation. Frame – 6 s, duration 3 min.

Supplementary Table

Table S1. Atomic coordinates of the model AML in the equilibrium ground S_0, first excited S_1 and transition states as well as in the conical intersection between the S_1 and S_0 states.

Equilibrium structure of the model AML, ground state S_0

N	1.939497295	-1.572571092	-0.987637363
C	0.830480859	-2.226392184	-0.493368047
C	-0.058067091	-1.534856877	0.268607441
C	0.070196393	-0.031922424	0.475106766
C	1.489605198	0.430601073	0.176985223
C	2.335843500	-0.315443150	-0.581839242
C	0.759818390	-3.678985352	-0.853920528
C	3.714012099	0.069577786	-1.026527853
H	2.600815914	-2.117953872	-1.516524829
C	-1.191535803	-2.244877254	0.868181703
H	-0.159456152	0.197016941	1.518160335
C	-0.940546568	0.723481368	-0.388694649
C	1.938868693	1.731232107	0.681907002
H	0.836534313	-4.305434890	0.038078830
H	-0.199513064	-3.924352639	-1.311952657
H	1.567091734	-3.944792214	-1.544352077
H	4.378589313	0.195382146	-0.168334311
H	4.132602166	-0.694263418	-1.690162234
H	3.706970759	1.029827371	-1.544467207
O	3.021414394	2.257927955	0.476419407

O	0.992535040	2.317281546	1.438348334
O	-1.456928789	-3.430553570	0.743450114
O	-1.948974743	-1.418989038	1.613315181
C	1.332465414	3.591677079	1.973362883
C	-3.081108484	-2.014923348	2.236887311
H	0.453141458	3.916209931	2.529000903
H	2.199726918	3.513753472	2.634020935
H	1.562674942	4.296727131	1.170851552
H	-3.578817463	-1.204292745	2.768314015
H	-3.747383379	-2.450974640	1.488521583
H	-2.770249798	-2.798975685	2.932114914
C	-0.858930518	0.597240883	-1.780896887
C	-1.736853017	1.245878295	-2.638404406
C	-2.739719174	2.054392067	-2.110783517
C	-2.849280052	2.202795786	-0.734709938
C	-1.958238099	1.543822119	0.112277686
H	-0.076017062	-0.034590388	-2.192826635
H	-1.639395649	1.121212442	-3.712710445
H	-3.436722366	2.570143938	-2.764561439
H	-3.623177492	2.828460880	-0.302719529
Cl	-2.179256168	1.795706220	1.824343000

Equilibrium structure of the model AML, first excited state S_1

N	2.020677901	-1.629334935	-0.835167933
C	0.880816344	-2.277664050	-0.460935785

C	-0.054758633	-1.545405728	0.298859601
C	0.149133812	-0.092352014	0.524135308
C	1.501412149	0.431219401	0.207218592
C	2.399281154	-0.350984203	-0.548236749
C	0.757486756	-3.696175616	-0.907096068
C	3.730190872	0.071543922	-1.074715395
H	2.671764862	-2.167462569	-1.392567152
C	-1.245472509	-2.203950880	0.811045355
H	-0.111480654	0.160687844	1.565981569
C	-0.889716670	0.686127217	-0.329851880
C	1.882262644	1.771087521	0.623794207
H	0.752023245	-4.387629974	-0.058875349
H	-0.183392450	-3.866432074	-1.436472423
H	1.588385040	-3.964313238	-1.570197806
H	4.433008131	0.291245820	-0.265196884
H	4.160903185	-0.714234716	-1.706465914
H	3.653932263	0.990962928	-1.660810085
O	2.960882677	2.310704882	0.393514063
O	0.912123674	2.381818341	1.336631806
O	-1.522801922	-3.391354888	0.666453569
O	-2.037136283	-1.362429675	1.509156941
C	1.207238539	3.703259140	1.769525561
C	-3.228829144	-1.933168361	2.033458512
H	0.313547801	4.041970199	2.293555817
H	2.071046913	3.707275976	2.439781125

H	1.421622684	4.351448642	0.915784637
H	-3.749389112	-1.112576645	2.527063218
H	-3.842807937	-2.351853853	1.231789208
H	-2.995789025	-2.726583597	2.748762796
C	-0.829740056	0.575970889	-1.725854433
C	-1.723273948	1.238353142	-2.550468222
C	-2.717882471	2.042814694	-1.989890546
C	-2.797907804	2.169244534	-0.611337835
C	-1.893906784	1.499148976	0.215889311
H	-0.055972338	-0.050358021	-2.162635633
H	-1.646087362	1.128807536	-3.627987135
H	-3.426598542	2.568879494	-2.621777450
H	-3.562062435	2.789452079	-0.154400834
Cl	-2.080029323	1.725286184	1.932271090

Transition state structure of the model AML, first excited state S_1

N	1.516167449	-1.621853632	-0.998525069
C	0.247215056	-1.933744310	-0.860603861
C	-0.382045638	-1.290070123	0.298305787
C	-0.088346905	0.154993750	0.462120079
C	1.410584418	0.390028596	0.321247888
C	2.173292704	-0.555148845	-0.335717105
C	-0.457901771	-2.803226328	-1.825314845
C	3.643642904	-0.537688031	-0.587745687
H	2.052238723	-2.066566901	-1.741511415

C	-0.840946508	-2.184610964	1.306559581
H	-0.409688977	0.465886651	1.458732321
C	-0.890990331	0.954866577	-0.571933395
C	1.972839715	1.616167854	0.860598775
H	-0.931577489	-3.623444029	-1.272541570
H	-1.263903890	-2.228840267	-2.301661719
H	0.202468328	-3.204127619	-2.599813360
H	4.146190241	-1.349516210	-0.046246141
H	3.865803740	-0.671889938	-1.657335716
H	4.068392852	0.405780941	-0.249232965
O	3.163007941	1.884772143	1.006444679
O	0.997703761	2.470578729	1.265163601
O	-0.825364152	-3.417016475	1.175041220
O	-1.297787835	-1.584362079	2.427093507
C	1.460105586	3.677912106	1.856487147
C	-1.778590513	-2.466665636	3.434131858
H	0.562323976	4.241793087	2.109725821
H	2.047651753	3.470843640	2.754949987
H	2.080597862	4.241423578	1.154675137
H	-2.128969731	-1.824908317	4.242389926
H	-2.597726181	-3.082338339	3.052780647
H	-0.980194833	-3.124647258	3.787625465
C	-0.409113041	1.127107843	-1.875890425
C	-1.148290395	1.785836320	-2.851299872
C	-2.399040248	2.308392683	-2.538314839

C	-2.896886639	2.166766292	-1.247521066
C	-2.153553199	1.492218229	-0.283438057
H	0.590580886	0.770126337	-2.109717881
H	-0.738154973	1.904594881	-3.849889823
H	-2.984478030	2.832829636	-3.287182987
H	-3.864801285	2.574786635	-0.976050654
Cl	-2.846070697	1.337199540	1.305450096

Conical intersection of the S_0 and S_1 states of the model AML

N	1.357412057	-1.743572863	-0.842059184
C	-0.015952705	-1.851455638	-0.841212829
C	-0.522099605	-1.206482339	0.377773152
C	-0.244913365	0.272018747	0.424556492
C	1.285176617	0.346387493	0.328766176
C	2.005918967	-0.667713733	-0.254661199
C	-0.761791566	-2.071474482	-2.102372993
C	3.498722902	-0.722530893	-0.360676799
H	1.905638995	-2.340308815	-1.444868650
C	-0.569108974	-2.006201733	1.587909564
H	-0.551327479	0.678966279	1.391294489
C	-0.977979631	1.045106249	-0.664563509
C	1.969565398	1.493712776	0.908456771
H	-0.876772512	-1.152129290	-2.700525590
H	-0.237565458	-2.800977573	-2.731523327
H	-1.760966769	-2.462601885	-1.898570226

H	3.956695415	-0.762836156	0.630502875
H	3.814263774	-1.603514906	-0.928739899
H	3.891963607	0.178592524	-0.834480550
O	3.177679970	1.665296181	0.988196079
O	1.091700305	2.412856149	1.374919999
O	-0.518150521	-3.227754036	1.605700341
O	-0.725293719	-1.267438036	2.708775943
C	1.679679506	3.570141010	1.958529192
C	-0.855541911	-2.019646921	3.910250800
H	0.844570941	4.192144744	2.280187981
H	2.305772009	3.298208639	2.812005623
H	2.294029330	4.102656762	1.228052771
H	-0.996542616	-1.285889778	4.703360320
H	-1.715419609	-2.692244286	3.852374807
H	0.043416124	-2.613847592	4.093144325
C	-0.311052210	1.615573020	-1.750388858
C	-0.989332591	2.315344941	-2.743262021
C	-2.370077457	2.463513709	-2.666645886
C	-3.062852329	1.911395196	-1.595489711
C	-2.367534227	1.212109979	-0.613386824
H	0.767846208	1.504047872	-1.804350034
H	-0.436330886	2.748057436	-3.571522420
H	-2.911932279	3.009766038	-3.432840266
H	-4.139340892	2.016782160	-1.511920397
Cl	-3.281681462	0.541212946	0.709882711

Table S2. Calculated absorption and emission spectra of AML and AML-5-COOH

AML			AML-5-COOH		
**λ (nm)**	***f***	**τ_{rad} (ns)**	**λ (nm)**	***f***	**τ_{rad} (ns)**
Absorption					
339	0.129		361	0.067	
			322	0.077	
Emission					
408	0.115	21.7	438	0.020	144.3

Synthesis of AML-5-COOH

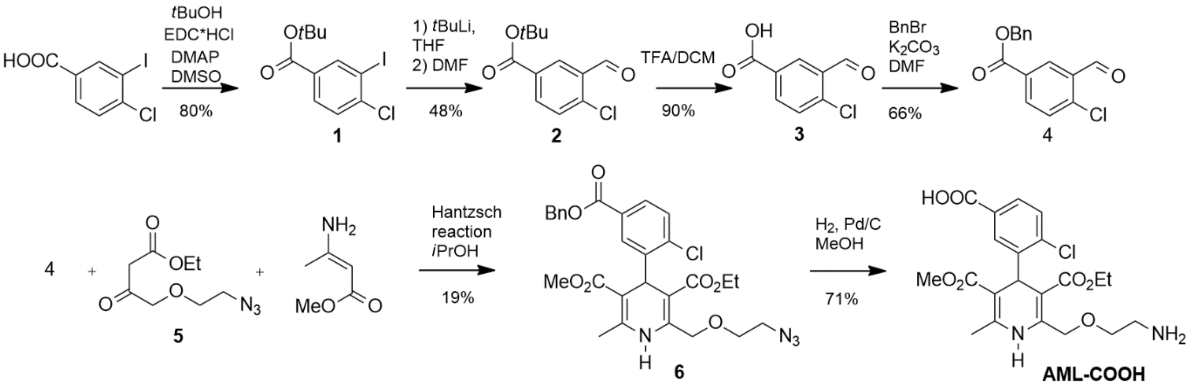

General materials and methods. Flash chromatography was performed using Biotage Isolera flash purification system with a cartridge and solvent gradient indicated. NMR spectra were recorded at ambient temperature with Agilent 400-MR spectrometer (MPI BPC Göttingen) at 400.06 MHz (^{1}H) and 100.60 MHz (^{13}C) and are reported in ppm. All ^{1}H spectra are referenced to tetramethylsilane (δ =0 ppm) using the signals of the residual protons of $CHCl_3$ (7.26 ppm) in $CDCl_3$, acetone-d_5 (2.05 ppm) in acetone-d_6, CHD_2OD (3.31 ppm) in CD_3OD or DMSO-d_5 (2.50 ppm) in DMSO-d_6. ^{13}C spectra are referenced to tetramethylsilane (δ = 0 ppm) using the signals of the solvent: $CDCl_3$ (77.16 ppm), CD_3OD (49.00 ppm) or DMSO-d_6 (39.52 ppm). Multiplicities of signals are described as follows: s = singlet, d = doublet, q = quartet, qd = quartet of doublets, m = multiplet or overlap of signals. Low resolution mass spectra (50 – 3500 m/z) with electro-spray ionization (ESI) were recorded on a Varian 500-MS spectrometer (Agilent) at MPI BPC Göttingen. High resolution mass spectra (ESI-HRMS) were recorded on a MICROTOF spectrometer (Bruker) equipped with ESI ion source (Apollo) and direct injector with LC autosampler Agilent RR 1200 at the Georg-August-Universität Göttingen. GC-MS spectra were recorded on Focus GC DSQ II (ThermoScientific) with Zebron ZB-5MS column; lenght=20m; id=0.18mm; film thickness = 0.18μm.

Compound 1 (4-chloro-3-iodobenzoic acid *tert*-butyl ester).

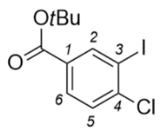

The flask was charged with 4-chloro-3-iodobenzoic acid (abcr, 5.1 g, 18 mmol,) in 130 ml DCM. Then *t*BuOH (1.7 g, 23 mmol), *N*-ethyl-*N'*-(3-dimethylaminopropyl)carbodiimide hydrochloride (Iris, 4.4 g, 23 mmol) and 4-(dimethylamino)pyridine (Acros Organics, 2.8 g, 23 mmol) were added gradually and the reaction mixture was stirred overnight at room temperature. Then it was washed with water (25 ml), sat. aq. $NaHCO_3$ (20 ml), 1M HCl (20 ml) and brine (2 ml). Afterwards

the organic layer was dried over Na_2SO_4, concentrated under reduced pressure and filtered through SiO_2 (100 g). Yield – 4.9 g (80%) of yellowish solid.

^{1}H NMR (400 MHz, $CDCl_3$) δ = 8.43 (d, J = 2.0 Hz, 1H, H-2), 7.88 (dd, J = 8.4, 2.0 Hz, 1H, H-6), 7.47 (d, J = 8.3 Hz, 1H, H-5), 1.58 (s, 9H, CH_3).

^{13}C NMR (101 MHz, $CDCl_3$) δ = 163.6 (CO), 143.0, 141.3, 131.8, 130.4, 129.1, 97.8, 82.2, 28.3 (CH_3).

EI–GC/MS (*m/z*): temp. 60°C – 270°C; ramp 30°C/min, hold for 5 min; 337.92 $[M]^+$ at t_R = 7.45 min.

Compound 2 (4-chloro-3-formyl benzoic acid *tert*-butyl ester).

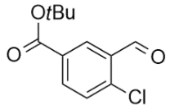

To a cooled to -78 °C solution of compound **1** (934 mg, 2.8 mmol) in anhydrous THF (21 ml) under Ar, 1.7 M *t*BuLi solution in pentane (4.9 ml, 8.4 mmol) was added rapidly in one portion. After stirring at -78 °C for 15 min, DMF (983 µl, 12.8 mmol) was added rapidly in one portion, and the resulting mixture was stirred at -78 °C for additional 5 min. Then the reaction mixture was slowly warmed up to room temperature and carefully quenched by addition of 4 ml PBS solution (pH 7.4) followed by addition of 1 M aq. HCl (8.5 ml). The organic phase was separated, the aqueous layer was extracted with EtOAc (2×3 ml). The combined organic solutions were dried over Na_2SO_4. The filtrate was evaporated, and the product isolated by flash column chromatography using SNAP Ultra 50g cartridge (gradient 0% – 20% ethyl acetate - hexane). Yield – 322 mg (48%) of yellowish solid.

^{1}H NMR (400 MHz, $CDCl_3$) δ = 10.47 (s, 1H, CH=O), 8.47 (dd, J = 2.2, 0.4 Hz, 1H), 8.12 (dd, J = 8.3, 2.2 Hz, 1H), 7.50 (dd, J = 8.4, 0.4 Hz, 1H), 1.59 (s, 9H).

^{13}C NMR (101 MHz, $CDCl_3$) δ = 189.1, 164.0, 141.7, 135.5, 132.4, 131.7, 130.8, 130.6, 82.4, 28.2.

Compound 3 (4-chloro-3-formyl benzoic acid).

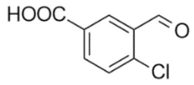

To a cooled to 0 °C solution of compound **2** (103 mg, 0.43 mmol) in DCM (1 ml) 1:1 mixture of TFA and DCM (1 ml) was added dropwise, and the reaction mixture was warmed up to room temperature and stirred for 1h. After diluting with DCM, washing with sat. aq. $NaHCO_3$ and drying over Na_2SO_4, the solvent was evaporated in vacuo. Yield – 79 mg (90%) of white solid.

^{1}H NMR (400 MHz, DMSO-d_6) δ = 10.33 (s, 1H), 8.38 (d, J = 2.2 Hz, 1H), 8.18 (dd, J = 8.3, 2.2 Hz, 1H), 7.64 (d, J = 8.3 Hz, 1H).

^{13}C NMR (101 MHz, DMSO-d_6) δ = 189.6, 167.4, 138.9, 135.8, 134.1, 131.8, 130.7, 130.5.

PCI–GC/MS (*m/z*): temp. 60°C – 270°C; ramp 30°C/min, hold for 5 min; 185.02 $[M]^+$ at t_R = 3.11 min.

Compound 4 (4-chloro-3-formyl benzoic acid benzyl ester).

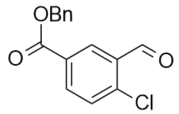

To a solution of compound **3** (97 mg, 0.52 mmol) in 5 ml DMF, 80 mg (0.58 mmol) K_2CO_3 was added and the suspension was warmed up to 40 °C. Then 98 mg (0.58 mmol) benzyl bromide was added, and the reaction mixture stirred at 40 – 42 °C for 3 h. After the solvent was evaporated *in vacuo*, the product was isolated by flash column chromatography (Biotage SNAP Ultra 10 g; gradient 3% to 20% MeOH in DCM). Yield – 94 mg (66 %) of yellowish oil. The product is unstable.

^{1}H NMR (400 MHz, $CDCl_3$) δ = 10.48 (s, 1H, CH=), 8.58 (dd, *J* = 2.2, 0.4 Hz, 1H, H-2), 8.20 (dd, *J* = 8.4, 2.2 Hz, 1H, H-6), 7.54 (dd, *J* = 8.4, 0.4 Hz, 1H, H-5), 7.48 – 7.31 (m, 5H, Bn), 5.38 (s, 2H, CH_2).

^{13}C NMR (101 MHz, $CDCl_3$) δ = 188.8 (CH=), 164.8 (CO), 142.3 (CO), 135.7, 135.5 (C-6), 132.6, 131.1 (C-5), 130.9 (C-2), 129.8, 128.8 (Bn), 128.7 (Bn), 128.5 (Bn), 67.5 (CH_2).

PCI–GC/MS (*m*/*z*): temp. 60°C – 270°C; ramp 30°C/min, hold for 5 min; 273.95 $[M]^+$ at t_R = 8.89 min.

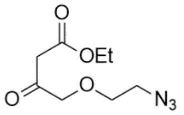

Compound 5 was obtained from 4-chloroacetoacetate (abcr, 800 mg, 4.86 mmol), 2-azidoethanol (abcr, 423 mg, 4.86 mmol) and NaH (60% in mineral oil, 200 mg, 5.21 mmol) in THF as described by Arrosmith et al.[8]

Compound 6. The dihydropyridine **6** was prepared from the keto ester **5** and aldehyde **4** by the method of Hantzsch.[8] A mixture of keto ester **5** (34 mg, 0.16 mmol), aldehyde **4** (40 mg, 0.14 mmol) and methyl 3-aminocrotonate (TCI, 18 mg, 0.16 mmol) in 2 ml 1:1 mixture *i*PrOH and EtOH was stirred at 95 ˚C for 16 h in a screw-cap tube. After the solvents were evaporated *in vacuo*, the product was isolated by flash column chromatography (Biotage SNAP Ultra 10 g; gradient 3% to 40% EtOAc in hexane). Yield – 15 mg (19 %) of yellow solid.

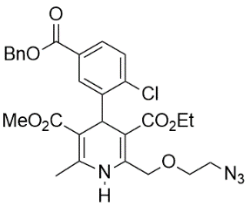

^{1}H NMR (400 MHz, $CDCl_3$) δ = 8.10 (d, *J* = 2.1 Hz, 1H, H_{Ar}, H-6), 7.74 (dd, *J* = 8.3, 2.1 Hz, 1H, H_{Ar}, H-4), 7.45 – 7.33 (m, 5H, $H_{Ar,}$ Bn), 7.31 (d, *J* = 8.3 Hz, 1H, H_{Ar}, H-3), 7.14 (s, 1H, NH), 5.44 (s, 1H, CH), 5.32 (s, 2H, CH_2-Bn), 4.75 (d, AB system, *J* = 16.2 Hz, 1H, CH_2O), 4.71 (d, AB system, *J* = 16.2 Hz, 1H, CH_2O), 4.03 (qd, *J* = 7.1, 4.3 Hz, 2H, $\mathbf{CH_2}CH_3$), 3.78 – 3.63 (m, 2H, NCH_2), 3.60 (s, 3H, OCH_3), 3.53 – 3.39 (m, 2H, CH_2), 2.34 (d, *J* = 0.5, 3H, CH_3), 1.15 (t, *J* = 7.1, 3H, $CH_2\mathbf{CH_3}$).

^{13}C NMR (101 MHz, $CDCl_3$) δ = 167.8, 167.0, 165.8, 146.2, 145.4, 144.7, 137.7, 136.1, 133.2, 129.6, 129.0, 128.7, 128.6, 128.5, 128.4, 128.3, 128.1, 128.1, 103.6, 101.2, 70.3, 68.2, 66.7, 60.0, 50.9, 50.9, 37.6, 19.4, 14.3.

ESI-MS, negative mode: *m*/*z* (rel. int., %) = 567 (100) $[M\text{-H}]^-$.

HRMS (*m*/*z*): $[M+H]^+$ calcd. for $C_{28}H_{29}ClN_4O_7$, 569.1798; found, 569.1789.

AML-5-COOH

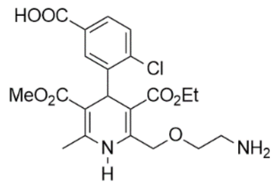

A 50 ml Schlenk-flask was evacuated and flushed with argon two times. Pd/C (17 mg; Merck, oxidized form) and MeOH (4 ml) were added, and the mixture was stirred vigorously under hydrogen to activate the catalyst. A solution of **6** (85 mg, 0.14 mmol) in 4 ml of MeOH was then added. The reaction mixture was stirred overnight at room temperature under H_2. Hydrogen was replaced with argon, and the mixture was filtered through Celite. The fillter cake was washed with MeOH. The solvents were evaporated *in vacuo*. The title compound was isolated by chromatography on SiO_2 (40 g) with CH_2Cl_2/MeOH mixture (5:1, 0.1% NH_4OH). Yield – 133 mg (95%) of light-brown solid. HPLC: t_R = 11.9 min (A/B: 10/90 – 100/0 in 25 min, 1.2 ml/min, 254 nm).

^{1}H NMR (400 MHz, CD_3OD) δ = 8.08 (d, J = 2.1, 1H, H-6), 7.73 (dd, J = 8.3, 2.1, 1H, H-4), 7.37 (d, J = 8.3, 1H, H-3), 5.49 – 5.42 (m, 1H), 4.78 (d, AB system, J = 14.3, 1H, CH_2O), 4.74 (d, AB system, J = 14.3, 1H, CH_2O), 4.05 (qd, J = 7.1, 3.3, 2H, **CH_2**CH_3), 3.85 – 3.71 (m, 2H, CH_2O), 3.59 (s, 3H, CH_3O), 3.25 (t, J = 5.0, 2H, CH_2N), 2.36 (s, 3H, CH_3), 1.17 (t, J = 7.1, 3H, CH_2**CH_3**).

^{13}C NMR (101 MHz, CD_3OD) δ = 169.5 (CO), 168.9 (CO), 168.4 (CO), 147.5 (C), 147.2 (C), 146.5 (C), 138.3 (C), 133.9 (CH, C-6), 130.9 (C), 130.6 (CH, C-3), 129.7 (CH, C-4), 103.8 (C), 103.6 (C), 68.7 (CH_2O), 67.8 (CH_2O), 61.1 (**CH_2**CH_3), 51.3 (CH_3O), 40.5 (CH_2N), 38.8 (CH), 18.8 (CH_3), 14.6 (CH_2**CH_3**).

ESI-MS, negative mode: m/z (rel. int., %) = 451 (50) $[M\text{-H}]^-$, 903 (100) $[2M\text{-H}]^-$.

HRMS (m/z): $[M+H]^+$ calcd. for $C_{21}H_{25}ClN_2O_7$, 453.1423; found, 453.1425.

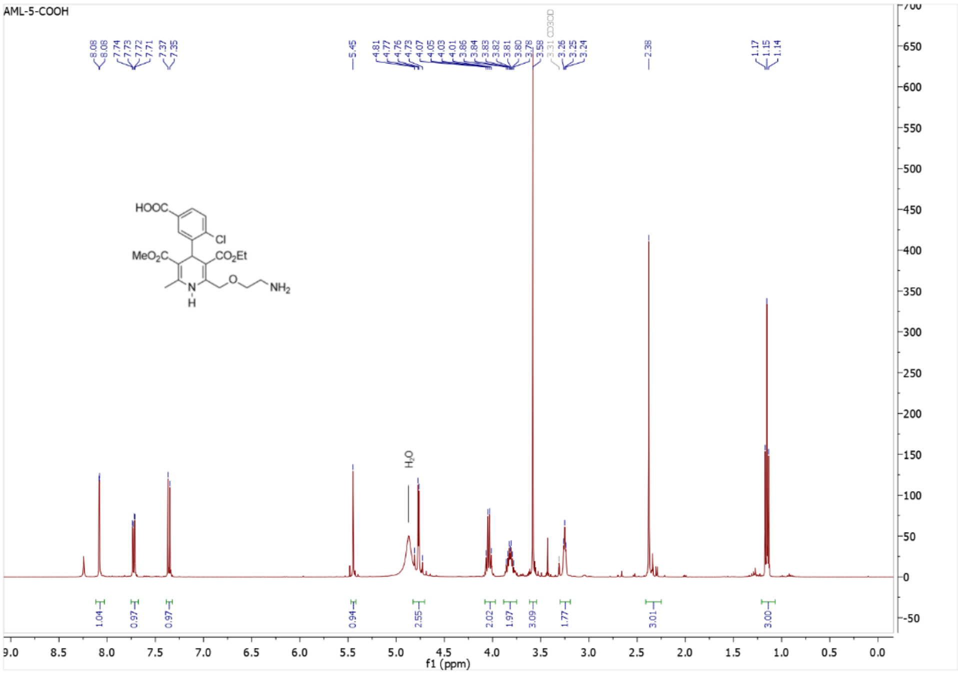

^{1}H spectrum of AML-5-COOH.

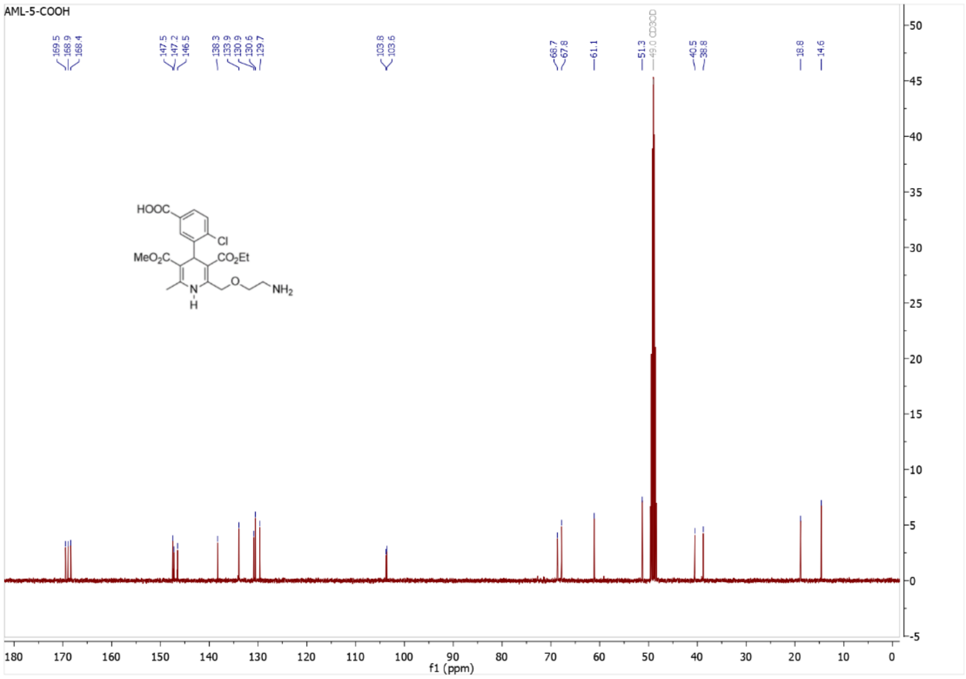

^{13}C spectrum of AML-5-COOH.

Abbrevations

A7r5 – Rat BDIX smooth muscle embryonic aorta cells
AML – Amlodipine
BSA – Bovine Serum Albumin
$Ca_v1.2$ – subunit of L-type voltage-dependent calcium channel
DAPI – 4′,6-Diamidin-2-phenylindol
DHP - 1,4-Dihydropyridine
DMAA – Dimethylacetamide
DMEM – Dulbecco's Modified Eagle Medium
DMF – Dimethylformamide
DMSO – Dimethyl Sulfoxide
FRET – Fluorescence Resonance Energy Transfer
GFP – Green Fluorescent Protein
HBSS – Hanks' Balanced Salt solution
HEPES – 4-(2-hydroxyethyl)-1-piperazineethanesulfonic acid
HEK-293 – Human Embryonic Kidney 293 cells
HEK-293 $Ca_v1.2$ – Human Embryonic Kidney 293 cells overexpressing $Ca_v1.2$
HL-1 – Atrial Muscle cells
HOMO – Highest Occupied Molecular Orbital
HSA – Human Serum Albumin
IC_{50} – Half maximal inhibitory concentration
K_d – Dissociation Constant
LCC – L-Type Ca^{2+}-channel
LFP – Laser-Flash Photolysis
LUMO – Lowest Unoccupied Molecular Orbital
PBS – Phosphate-Buffered Saline
(*S*)-AML – (*S*)-Amlodipine
SDS – Sodium Dodecyl Sulfate
THF – Tetrahydrofuran

References

[1] Lax, M. (1952) The Franck-Condon Principle and Its Application to Crystals, *The Journal of Chemical Physics 20*, 1752-1760.
[2] Heller, E. J. (1981) The semiclassical way to molecular spectroscopy, *Accounts of Chemical Research 14*, 368-375.
[3] Yurenev, P. V., Kretov, M. K., Scherbinin, A. V., and Stepanov, N. F. (2010) Environmental Broadening of the CTTS Bands: The Hexaammineruthenium(II) Complex in Aqueous Solution, *The Journal of Physical Chemistry A 114*, 12804-12812.
[4] Kretov, M. K., Iskandarova, I. M., Potapkin, B. V., Scherbinin, A. V., Srivastava, A. M., and Stepanov, N. F. (2012) Simulation of structured 4T1→6A1 emission bands of Mn2+ impurity in Zn2SiO4: A first-principle methodology, *Journal of Luminescence 132*, 2143-2150.
[5] Kretov, M. K., Scherbinin, A. V., and Stepanov, N. F. (2013) Simulating the structureless emission bands of Mn2+ ions in ZnCO3 and CaCO3 matrices by means of quantum chemistry, *Russian Journal of Physical Chemistry A 87*, 245-251.
[6] Rukin, P. S., Freidzon, A. Y., Scherbinin, A. V., Sazhnikov, V. A., Bagaturyants, A. A., and Alfimov, M. V. (2015) Vibronic bandshape of the absorption spectra of dibenzoylmethanatoboron difluoride derivatives: analysis based on ab initio calculations, *Physical Chemistry Chemical Physics 17*, 16997-17006.
[7] Richter, K. N., Revelo, N. H., Seitz, K. J., Helm, M. S., Sarkar, D., Saleeb, R. S., D'Este, E., Eberle, J., Wagner, E., Vogl, C., Lazaro, D. F., Richter, F., Coy-Vergara, J., Coceano, G., Boyden, E. S., Duncan, R. R., Hell, S. W., Lauterbach, M. A., Lehnart, S. E., Moser, T., Outeiro, T. F., Rehling, P., Schwappach, B., Testa, I., Zapiec, B., and Rizzoli, S. O. (2018) Glyoxal as an alternative fixative to formaldehyde in immunostaining and super-resolution microscopy, *Embo Journal 37*, 139-159.
[8] Arrowsmith, J. E., Campbell, S. F., Cross, P. E., Stubbs, J. K., Burges, R. A., Gardiner, D. G., and Blackburn, K. J. (1986) Long-acting dihydropyridine calcium antagonists. 1. 2-Alkoxymethyl derivatives incorporating basic substituents, *Journal of Medicinal Chemistry 29*, 1696-1702.
